# Global Gene Expression Analysis of the Interaction between Cancer Cells and Osteoblasts to Predict Bone Metastasis in Breast Cancer

**DOI:** 10.1371/journal.pone.0029743

**Published:** 2012-01-03

**Authors:** Michal Rajski, Brigitte Vogel, Florent Baty, Christoph Rochlitz, Martin Buess

**Affiliations:** 1 Division of Medical Oncology, Department of Biomedicine, Basel University, Basel, Switzerland; 2 Institute of Physiology, University of Zürich, Zürich, Switzerland; 3 Division of Pneumology, Cantonal Hospital St. Gallen, St. Gallen, Switzerland; 4 Division of Medical Oncology, Basel University Hospital, Basel, Switzerland; 5 Division of Oncology, St. Claraspital, Basel, Switzerland; Chang Gung University, Taiwan

## Abstract

**Background:**

Bone metastasis is a main cause of morbidity in breast cancer. Since breast cancer is a heterogeneous disease, the interactions of cancer cells with the skeletal host cells might also be diverse. We hypothesized that gene expression signatures induced by heterotypic interaction of breast cancer cells and osteoblasts might be of clinical relevance.

**Methodology/Principal Findings:**

We established an ex vivo co-culture model using benign breast epithelial cells or a panel of 5 malignant breast epithelial cells in combination with primary human osteoblasts and determined associated gene expression changes with HEEBO microarrays. Pretreatment gene expression profiles of 295 early stage breast cancers published from the Netherlands Cancer Institute with a median follow up of 12.6 years allowed evaluating in vitro effects in the in vivo situation.The effects of the interaction between osteoblasts and breast cancer cell lines of different origin were very heterogeneous. Hs578T cells started to proliferate in co-culture with osteoblasts, SKBR-3 induced a TGF-β response and MDA-MB231 cells showed two distinct sets of up-regulated genes: A set of interferon response genes associated with an up-regulation of STAT1 was in vivo remarkably coherent providing a basis for segregation of tumors into two groups. In a uni-variate analysis, early stage tumors with high expression levels (n = 136) of this gene set had a significantly lower overall survival rate (p = 0.005) (63% at 10 years) than tumors with low expression levels (n = 159) (overall survival: 77% at 10 years). The second gene set was associated with IL-6 and did not significantly change the overall survival rate (p = 0.165), but was significantly associated with a shorter time to bone metastasis (p = 0.049; 74% vs. 83% at 10 years).

**Conclusion/Significance:**

An IL-6 gene expression pattern induced by heterotypic interaction of breast cancer cells with osteoblasts in vitro is associated with a higher rate of bone metastasis in vivo.

## Introduction

Bone is the most common site of breast cancer metastasis, and this type of metastasis is frequently the main cause of morbidity in patients with breast cancer. Bone metastases are associated with pain, decreased mobility, fractures, neurologic compromise and symptoms of hyper-calcemia [1]. Approximately 70% of patients with metastatic breast cancer develop bone metastases as their disease progresses [2]. Cancer metastasis to distant organs is modulated by inherent properties of metastatic cancer cells and the host microenvironment encountered by those cancer cells. During the process of metastasis, cancer cells may acquire additional capacity for progression under the influence of the host microenvironment, and conversely, the host environment may be changed by the presence of cancer cells. The reciprocal interactions between cancer cells and the host environment are critical for the progression of metastasis to target organs; in fact, this concept was stated by Paget a hundred years ago as the “seed and soil” hypothesis [3].

Metastasis formation in the bone is a complex process that requires cooperative reciprocal interaction between tumor cells and the cellular environment of the bone, which includes osteoclasts and osteoblasts [4]. These interactions involve a plethora of soluble and cellular components that interplay in a process of coordinated expression and mutual signaling [5]. Multiple single factors have been described in numerous reports to be involved in bone metastasis formation; for example, growth factors, such as bone morphogenetic proteins (BMPs), transforming growth factor β (TGF-β), insulin-like growth factors (IGFs), and fibroblast growth factors (FGFs) have been shown to play important roles. In addition, previous studies have indicated the importance of membrane-bound molecules, such as cadherins, and extracellular matrix components, including laminin, collagens and matrix metalloproteinases (MMPs) [5]. However, it is likely that there are other factors that remain unidentified. The interplay between the various factors and their combined effects on bone metastasis also remain to be further characterized.

Like primary tumors, metastases are not merely aggregates of malignant epithelial cells; instead, in many respects, they are organ-like structures that include host stromal cells, such as fibroblasts, inflammatory cells, endothelial cells, which form the vasculature and the parenchymal cells of the target organ, like the osteoblasts that form the bone. The malignant cells themselves intermingle and interact with all of these cell types. There is growing evidence that besides the cellular processes within the tumor cells, a relevant contribution to tumor progression is provided by the cells of the tumor microenvironment. On the molecular level, genomic gene expression studies analyzing many different carcinomas have illustrated in detail the complexity of the tumors and the diversity of the associated non-epithelial cell types [6]–[9]. Inductive interactions between these different cell types have been shown to play not only a morphogenetic role but also an important mechanistic role in the pathogenesis and progression of malignancy [10]. Thus far, osteoblasts have been mainly viewed in the context of bone formation and skeletal remodeling [11]. However, relatively little is known about the paracrine effects of these tumor-osteoblast interactions. In breast cancer, it was commonly thought that during osteolytic metastasis, when the bone is mainly degraded by the activation of osteoclasts, the signal exchange between cancer cells and osteoclasts (e.g., osteoprotegerin and RANKL) plays the major role and that the bisphosphonates hinder osteolytic bone degradation, thereby releasing matrix bound growth factor, which stimulates metastatic progression [12]. However, substantial evidence has indicated that the osteoblasts also have a role in this interplay and that some effects of these agents could be due to the disruption of the paracrine metastasis-promoting signaling that occurs as a result of the interaction between the cancer and osteoblast cells [4]. Such reciprocal inductive signaling has been well known from the developmental biology perspective and has again attracted special attention with the development of the concept of cancer stem cells and their stem cell niches [13]. In this respect, osteoblastic cells are of special interest because breast cancer cells display long latency periods in the bone before metastasis formation [14]. This observation indicates that osteoblasts and their precursors, the mesenchymal stem cells, might form a niche for metastasis-initiating cells [15]. The ability of osteoblasts to regulate the hematopoietic stem cell niche would support this hypothesis. Whether this niche [16] can be influenced by pharmacologic intervention e.g. zolendronic acid or denosumab and hinder metastatic relapse in the adjuvant situation is a matter of debate [17]. Therefore, characterizing heterotypic cell-cell interaction effects on a global gene expression scale might help to better understand the currently used agents and eventually lead to the identification of novel targets that could be used to interrupt these paracrine stimulatory signaling pathways.

Breast cancer is a heterogeneous disease, which implies that the tumor-osteoblast interactions might also be diverse. Tumor-osteoblast interactions have not yet been well characterized on a genome-wide scale, and they have not been compared among different tumor subtypes. We recently used an approach of *in vitro* co-culture experiments to characterize heterotypic interactions with DNA microarrays to systematically describe the global effects that the tumor-fibroblastic stroma interaction has on gene expression. We identified a strong induction of the interferon response by specific tumor cells that were co-cultured with a diverse set of fibroblasts, and this response correlated with a subset of breast cancers that had an unfavorable prognosis *in vivo* [10]. Furthermore, we studied tumor-endothelial interactions and realized that the interaction of endothelial cells with a subset of CD44+/CD24− breast cancer cell lines induces a signature of “tumor-endothelial cell-induced M phase/cell cycle” genes, which are associated with an unfavorable outcome in human breast cancer. [18].

In this study we investigated the effects of heterotypic interaction between different breast cancer cell lines and and human osteoblasts on the global gene expression programs to obtain clues to the signaling mechanisms that are involved in bone metastasis. We speculated that the interactions between tumor and osteoblast cells lead to the induction of gene expression signatures that are clinically relevant. These interactions might account for a significant proportion of the unexplained information in the gene expression data from various tissue specimens. Given the evidence that interactions between cells can play critical roles in tumor progression, these data might be even more meaningful than prominent expression patterns, which are driven by the proportional representation of a given cell type in a tissue. With this approach we identified different interaction patterns and demonstrated that the IL-6 gene expression signature is predictive for the development of bone metastasis.

## Methods

The study was approved by the ethics committee: Ethikkommission beider Basel, Switzerland (approval No. 271/05) including the analysis of publicly available datasets of breast cancer patients. For the retrospective analysis of datasets, the ethics committee waived individual consent of these patients whose data were analyzed anonymously.

### Cell culture

Human mammary epithelial cells (HMECs) (Cambrex Bio Science Walkersville, Walkersville, MD) were expanded in mammary epithelial basal medium that was supplemented with bovine pituitary extract, human EGF, insulin and antibiotics (Clonetics, Cambrex Bio Science Walkersville, Walkersville, MD). MCF-7, T47D, MDA-MB-231, SKBR-3, and Hs578T cells (ATCC, Atlanta, GA) were propagated in DMEM with 4.5 g/l glucose (Gibco, Grand Island, NY) that was supplemented with 10% FBS (HyClone, Logan, UT), glutamine, 100 U/ml penicillin and 100 µg/ml streptomycin (Gibco, Grand Island, NY). Normal human osteoblasts (NHOst) (Lonza Walkersville, Walkersville, MD) were expanded in Clonetics Osteoblast Basal Medium (OBM™) (Lonza Walkersville, Walkersville, MD) supplemented with osteoblast growth medium (OGM™) SingleQuots® (Lonza Walkersville, Walkersville, MD) and 10% FBS. For the co-culture experiments and control cell cultures, cells were cultivated for 48 hours at a total density of 30,000 cells/cm^2^ (15,000 tumor cells/cm^2^ and 15,000 normal human osteoblast cells/cm^2^) in Clonetics Osteoblast Basal Medium (OBM™) supplemented with 0.2% FBS without any further additives. This medium served as a good universal medium for all of the cells in this study.

### Proliferation assays

#### Direct cell counting

For cell counting, pre-starved cells were plated in quadruplicate in 24-well plates at a density of 8500 cells/cm^2^. After 24 and 48 hours, the cells were trypsinized and re-suspended in 0.2 ml FACS buffer that contained 0.5% BSA and 2 mM EDTA in PBS. The total cell number was determined using a cell counter.

#### Comparison of cell proliferation in response to different conditioned media

To obtain the conditioned medium, 10e6 Hs578T or NHOst cells were extensively washed to avoid transfer of any stimuli from the regular cell growth media. The cells were kept at a density of 50,000 cells/cm^2^ in osteoblast basal medium that contained 0.2% FBS without additional supplements for 48 hours. The medium was then aspirated and filtered through a 0.2 nm pore filter. In parallel, in a 12-well plate, 34,000 NHOst or Hs578T cells/well were starved for 24 hours in osteoblast basal medium that contained 0.2% FBS without supplements. For the stimulation experiments, NHOst or Hs578t cells were washed once with PBS and incubated for 24, 48 or 72 hours in 50% osteoblast basal medium that contained 0.2% FBS and 50% conditioned medium. Osteoblast basal medium with 0.2% FBS was used as a negative control (vehicle medium), NHOst cell culture supernatant that was diluted 1∶2 in vehicle medium was used as an autologous medium control, and full osteoblast basal medium supplemented with Single Quots as described above was used as a positive control. To determine cell proliferation in response to stimulation with conditioned medium, the cells were directly counted after 24, 48 and 72 hours using a cell counter. Experiments were done in triplicate.

### Real-time quantitative PCRs

To determine IL-6 mRNA levels, cells were kept under the conditions described above and harvested after 48 hours. For real-time quantitative PCR, cDNA was prepared with the Maxima First Strand cDNA Synthesis Kit (Fermentas #K1641, Fermentas International, Inc., Burlington, Ontario, Canada). For amplification, we used the MESA GREEN qPCR MasterMix Plus (Eurogentec # RT-SY2X-20, Eurogentec S.A., Belgium) with 300 nM of the following primers: IL-6 Forward: 5′ - TAC CCC CAG GAG AAG ATT CC - 3′, Reverse: 5′ - GCC ATC TTT GGA AGG TTC AG - 3′; hRPL-19 Forward: 5′- GCC CAT CTT TGA TGA GCT TC - 3′, Reverse: 5′ - GTG GCA AGA AGA AGGF TCT GG - 3′. All primers were designed with PrimerBank [19]. Reactions were detected on an Applied BioSystems 7000 real-time PCR instrument.

### ELISAs

For IL-6 enzymatic linked immunosorbent assays, we used the Human IL-6 Quantikine Immunoassay (R&D Systems, Abingdon, OX14 3NB United Kingdom) according to the manufacturer's recommended instructions.

### RNA isolation and amplification

For microarray experiments, single-cell cultures or co-cultures were incubated for 48 hours to allow reciprocal signal exchange. After discarding the culture medium and washing the cell layer once with PBS, total RNA was isolated by lysing the cells in the culture dish with RLT buffer (Qiagen, Valencia, CA) and extracting the RNA with the RNeasy® Mini Kit (Qiagen). Five hundred nanograms of total RNA were amplified using the Message Amp™ II aRNA Kit (Ambion, Austin, TX). The RNAs and the amplification products were checked for integrity by electrophoresis in a 1% agarose gel in MOPS buffer.

### cDNA Microarrays and Hybridization

For global gene expression analysis, we used HEEBO microarrays. The HEEBO microarrays consist of 44,544 70-mer probes that included the following: (a) constitutive exonic probes (30,718), (b) alternatively spliced/skipped exonic probes (8,441), (c) non-coding RNA probes (196), (d) BCR/TCR genic/regional probes (372), (e) other probes (843), and (f) controls and empty spots as negative controls for background fluorescence (3974). HEEBO microarrays were produced at the Stanford Functional Genomic Facility (Stanford, USA). Complete details regarding the clones on the arrays may be found at: http://www.microarray.org/sfgf/heebo.do.

For microarray experiments, 8 µg amplified RNA (aRNA) was mixed with doping controls. Samples were vacuum dried, resolved in coupling buffer and labeled with Cy5 dye. Labeled samples were pooled with equal amounts of reverse colored Cy3-labeled amplified reference RNA from Stratagene (Stratagene, CA, USA). The labeled aRNA was purified with the AminoAllyl MessageAmp ™ II aRNA Amplification Kit (Ambion) according to the manufacturer's suggested protocol and fragmented using fragmentation reagents (Ambion). The fragmented probes were added to a hybridization buffer containing Cot/PolyA/tRNA (0.05 µg/uL each), 0.3% SDS, and 3.3× SSC and supplemented with HEPES buffer. Following a denaturing step at 100°C, the probe was placed on the microarray for competitive hybridization. After 18 hours, slides with hybridized probes were sequentially washed and immediately dried in an ozone-free environment.

### Data Analyses and Clustering:

Array images were scanned using an Axon Scanner 4000B (Axon Instruments, Union City, CA), and image analysis was performed using Genepix Pro, version 5.0 3.0.6.89 (Axon Instruments). The raw data files were stored in the Stanford Microarray Database [20] . Data are expressed as the log_2_ ratio of fluorescence intensities of the sample and the reference for each element on the array. In addition, MIAME compliant microarray data were submitted to Gene Expression Omnibus (GEO) [21] and are accessible through GEO (http://www.ncbi.nlm.nih.gov/geo/query/acc.cgi?acc=GSE29036).

The (Cy5/Cy3) ratio is defined in the Stanford Microarray Database (SMD) as the normalized ratio of the background-corrected intensities. Spots with aberrant measurements that were due to obvious array artifacts or poor technical quality were manually flagged and removed from further analysis. A filter was applied to omit measurements where the fluorescent signal from the DNA spot was less than 50% above the measured background fluorescence that surrounded the printed DNA spot in either the Cy3 or Cy5 channel. Genes that did not meet these criteria for at least 80% of the measurements across the experimental samples were excluded from further analysis. Valid data were filtered to exclude elements that did not have at least a 3-fold deviation from the mean 2 samples. Data were analyzed using unsupervised hierarchical clustering [22] (average linkage, un-centered correlation) and were displayed with the Treeview software (http://rana.lbl.gov/EisenSoftware.htm).

### GO::TermFinder

GO::TermFinder is comprised of a set of object-oriented Perl modules for accessing Gene Ontology (GO) information to evaluate and visualize the collective annotation of a list of genes to GO terms [23]. It can be used to draw conclusions from microarrays by calculating the statistical significance of each annotation.

### Determination of the heterotypic interaction effect on gene expression

To facilitate the identification of heterotypic interaction effects on global gene expression in a mixed co-culture experiment, gene expression data were normalized based on the proportional contribution of each cell type to transcript abundance. Given that the average gene does not change due to heterotypic interaction and that there are simple additive effects to be considered, a linear regression fit was used for normalization. To determine the contribution of each cell type to the combined gene expression pattern in the linear regression model, the expression levels of the monocultures were considered the predictors, and the expression levels of the co-culture were considered the response.

Specifically, a set of equations (1-n) was established (one per gene): e_n_^co-culture^ = ((a×e_n_^monoculture1^)+((1-a)×e_n_^monoculture2^))×I_n_, where e represented the expression level of the gene, a represented the proportional contribution of mRNA from the respective monoculture, n represented the number of genes measured on the microarray and I represented the interaction coefficient. We assumed that the average gene is not influenced by heterotypic interaction in the mixed co-culture, which is represented as I = 1. Since the dataset over e_1-n_ is skewed, a linear regression fit was empirically identified based on Gamma errors and identity link as a good model to calculate a. The equations 1-n can then be solved for I_1-n_ , which results in a profile of interaction effects for the genes_1-n_. These interaction effects can be analyzed in much the same way as conventional gene expression measurements.

### Human breast cancer dataset

The dataset for breast cancer contained 295 tumors that were analyzed on a 25,000 spot oligonucleotide array [24]. In brief, patients were diagnosed and treated at the Netherlands Cancer Institute (NKI) for early stage breast cancer (stage I and II) between 1984 and 1995. The clinical data were updated in January 2005. The median follow-up for patients who are still alive is 12.3 years.

The “IL-6 gene signature” consisting of 72 gene IDs (63 unique) was mapped to the spots on the NKI array using Unigene build no. 184 (released on 06-09-2005). As a result, we obtained 28 unique spots for each of 295 patients. The “interferon response genes regulated by STAT1 signature” consists of 94 (62 unique) genes. A procedure similar to that of the “IL-6 gene signature” was applied, resulting in 26 unique spots for each patient. Expression measurements for each gene were mean centered. The resulting dataset was subjected to hierarchical clustering [22] with average linkage clustering and displayed with Treeview (http://rana.lbl.gov/EisenSoftware.htm).

Distant metastasis was analyzed as a first event only (Distant Metastasis-Free Probability: DMFP). If a patient developed a local recurrence, an axillary recurrence, a contra-lateral breast cancer, or a second primary cancer (except for non-melanoma skin cancer), she was censored at that time, and the subsequent distant metastases were not analyzed. This decision is based on the theoretical possibility that the locally recurrent or second primary cancers could be a source for distant metastases. An ipsilateral supra-clavicular recurrence was soon followed by a distant metastasis in all but one patient. Thus, an ipsilateral supra-clavicular recurrence was considered to be the first clinical evidence of metastatic disease for this analysis, and patients were not censored at the time of ipsilateral supra-clavicular recurrence. Overall survival was analyzed based on death from any cause, and patients were censored at the last follow-up. Data with time to bone metastasis were taken from the paper by Bos et al. [25].

To stratify patients based on differential IL-6 gene expression, we used a threshold above or below the value of a reference pool of mRNA. There were 178 patients that expressed the IL-6 gene at levels below the reference and 117 patients that expressed the IL-6 at levels above the reference gene. Data with time to bone metastasis were taken from the paper by Bos at al. [25].

### Centroid correlation

The method of calculating the centroid for each patient was described previously [26],[27]. The centroids were profiles that consisted of the average gene expression value for each of the patients. Briefly, the centroids for the genes that represented the “IL-6 gene signature” and other previously described signatures were calculated based on the NKI dataset. To test the similarity between the signatures, the correlation between values of different centroids was checked for each patient. The correlation was calculated using Pearson's correlation coefficient using the R software [28].

### General statistic methods

Normally distributed data were analyzed using Student's *t*-test. Differences were considered as statistically significant when p<0.05. All statistical tests were performed using the R statistical software (version 2.10.1) [28]. Survival curves were obtained using the Kaplan-Meier estimator and Cox proportional hazards regression models were fitted (R package “survival”).

## Results

### Design of a tumor-osteoblast co-culture model

**Figure 1:**
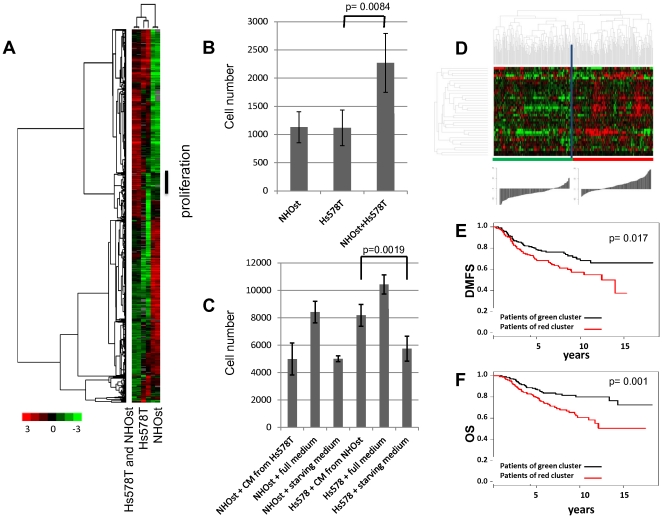
Effects of a heterotypic interaction between normal human osteoblasts and breast cancer cells. (**A**) Biologically independent replicates of NHOst, Hs578T, and the mixed co-culture of NHOst and Hs578T were kept for 48 hours at low serum conditions and characterized by DNA microarray profiling. We performed hierarchical clustering of 1923 elements that display a greater than 3-fold variance in expression in more than 2 different experimental samples. Genes are represented in rows and experiments in columns. Unsupervised hierarchical clustering of the experiments grouped the biological replicates together. The vertical black bar marks a cluster of genes that were higher expressed in all co-cultures compared to both monocultures, which indicated that they were induced by the heterotypic interaction. Further analysis of these genes revealed that they were specific for proliferation and mitosis. (**B**) The proliferation rate of NHOst and Hs578T cell monocultures and of their 1∶1 co-culture was determined by measuring increases in cell number by direct cell counting. Quadruplicates of pre-starved cells were plated at a density of 8500 cells/cm^2^ and after 48 hours the cell number was determined using a cell counter All figures represent averages from four replicates, and error bars denote standard deviation. The increase in cell number of the co-culture is significantly higher than the increase in both of the monocultures (p = 0.0084, un-paired, two-tailed *t*-test). (**C**) Hs578T cells and NHOst cells were incubated for 24 hours with conditioned media (CM) from NHOst cells or Hs578T cells, respectively, and compared to a negative control of the same cells incubated with autologous medium. All experiments were performed in triplicates. After 24 hours cell numbers were measured by the cell counting with FACS. (**D**) The expression values of the genes in the “tumor-osteoblast cell-induced M phase/cell cycle” gene signature were extracted from a published expression study of 295 early stage breast cancers from the Netherlands Cancer Institute. Genes and samples were organized by hierarchical clustering. The tumors were segregated into two groups that were defined by high or low expression levels of the 36 genes matching the proliferation gene cluster. The histogram below the heat map represents the differences in the sums of log2 ratios among groups. Based on the distributions, the sums of the log2 ratios for the “proliferation” transcripts were over-expressed in the majority of the cases in the right branch of the cluster compared to the left branch of the cluster (32/113 versus 105/45 scores below/above reference zero value, respectively). (**E+F**) Correlation of the “proliferation” gene signature with distant metastasis-free survival (DMFS) (E) and overall survival (OS) (F). Kaplan-Meier curves for the clinical outcomes of the indicated tumors that exhibited high (red curve) and low (black curve) expression of the “proliferation” signature are shown.

As a model for investigating changes in gene expression in response to epithelial-osteoblast interactions in bone metastases of breast carcinomas, cells that represented either benign or malignant epithelial cell compartments and cells that represented skeletal compartments were examined in an *in vitro* mixed co-culture setting. These two types of cells were co-cultivated for 48 h in a low-serum medium [0.2% fetal bovine serum (FBS)] to allow reciprocal signal exchange with minimal background from undefined molecular signals that are inherent in fetal bovine serum. We examined the effects of co-cultivation on each cell pairing in two independent biological replicates. The gene expression profiles of the co-cultures were compared to the expression profiles of the corresponding cells that were kept in monoculture using HEEBO microarrays that contained 44,544 70-mer oligonucleotide probes. To establish this experimental approach, we first focused our experiments on the breast cancer cell line Hs578T, the primary human osteoblast cells NHOst and the co-culture of these two cell types. The data that passed our filter of data quality and a filter for data distribution were organized using unsupervised hierarchical clustering of the replicate experiments to provide an overview of the effects on global gene expression (**Figure 1A**). Biological replicates clustered together and the differential gene expression patterns of NHOst and Hs578T cells confirmed that these cell types have distinct default gene expression patterns. In the co-culture, most genes displayed intermediate expression levels, which closely approximated the proportionally weighted average of their expression levels in the two cell types in monoculture. Despite setting up the co-culture with equal cell numbers of Hs578T and NHOst cells, the gene expression pattern after co-cultivation was dominated by the pattern of the Hs578T cells. However, one set of genes (86 gene IDs, 80 unique genes) showed a consistent increase in transcript abundance in the co-culture when compared to either monoculture, which suggested that the induction of these genes was an effect of the co-cultivation of the breast cancer and osteoblast cells.

Interestingly, out of 46 genes within this gene set identified using GO::TermFinder [23] 17 (37%) were involved in nucleic acid metabolic processes, as determined by GO-terms; PSMC3IP, HMGB1L1, SFPQ, MCM3, PPP2CA, ORC6L, ABCE1, PA2G4, CHEK1, SF3A3, PPIH, CDC7, PCNA, MAGOH, RAN, NOP58, and HMGB2 (**Figure S1**). The frequency of the genes that are involved in this function was significantly enriched (p = 0.049) compared to the background of 922 genes passing the data quality and data distribution filters, as shown in the heat map of Figure 1 and present in GO::TermFinder database. Out of these 17 genes, 5 (MCM3, ORC6L, CHEK1, CDC7, and PCNA) are directly annotated as involved in DNA replication. The up-regulation of these gene sets suggests proliferation of the co-culture of the breast cancer cells (Hs578T) and osteoblast cells.

As implied by the higher expression of the “proliferation” gene signature, the proliferation rate, as determined by direct cell counting over time, was significantly higher in the co-culture of Hs578T and NHOst cells than in isolated Hs578T or NHOst cell monocultures (p = 0.0084, un-paired, two-tailed *t*-test) (**Figure 1B**).

We speculated that specific factors secreted by Hs578T cells might increase the growth of the NHOst cells or vice versa. Towards this aim, we reciprocally incubated one cell type in conditioned medium from the other cell type. In fact, when the conditioned medium from the NHOst cells was applied to Hs578T cells, the Hs578T cells proliferated at significantly higher rate than when incubated in autologous medium, as measured by relative increases in cell numbers (p = 0.0019, un-paired, two-tailed *t*-test) (**Figure 1C**). Conversely, conditioned medium from Hs578T cells did not stimulate the proliferation of NHOst cells. This result indicates that a stimulatory factor secreted by the osteoblast cells creates an environment that induces tumor cell proliferation.

We investigated the effects on global gene expression in response to heterotypic cell-cell interaction as a simple, controlled, *ex vivo* model of tumor-osteoblast cell interaction. We reasoned that identifying and characterizing gene expression patterns that were characteristically induced by the interaction between specific pairs of cells in culture might enable us to recognize and interpret specific features in the expression profiles of human cancers that represent similar interactions between tumor and osteoblasts *in vivo*. To verify the relevance of our *in vitro* experiments, we examined the expression levels of the genes found to be induced in the co-culture of Hs578T and NHOst cells in the global gene expression data of early stage breast cancer biopsies from 295 patients from the Netherlands Cancer Institute (NKI), which is publicly available information [24]. The cluster of 36 (36 present in NKI out of 80 unique) “proliferation” genes showed strikingly coherent variation in expression among these patients with cancer, which enabled these patients to be divided into two groups (**Figure 1D**). One group had a relatively high expression level of the “proliferation” genes, and the other group was characterized by a relatively low expression level. To visualize the difference in expression levels between groups, we calculated the scaled gene expression score for each tumor by summing up the log2 based gene expression ratios of the 36 genes building the proliferation cluster measured in each of the 295 breast cancer samples while taking into account the direction of the gene expression. The distributions of the scores for the two main branches were significantly different (p<2.2e-16, un-paired, two-sided *t*-test). The left branch (average score; −3.93+/−5.3) and the right branch (average score; 3.8+/−5.4) are shown in the histogram below the heat map in Figure 1D. Based on the distributions, the sums of the log2 ratios for the “proliferation gene” transcripts were over-expressed in the majority of the cases in right hand side branch of the cluster, as compared to left hand side branch of the cluster (32/113 versus 105/45 scores below/above reference zero value, respectively). To simplify further discussion, we named this signature the “proliferation” signature.

To assess the potential biological relevance of this gene expression signature, distant metastasis-free survival and overall disease-specific survival were compared between the two groups. Early stage tumors with high expression levels (n = 145) of this particular gene set had a significantly lower distant metastasis-free survival (p = 0.017; 56.9% at 10 years) and overall survival rate (p = 0.001; 60.5% at 10 years) than tumors with low expression levels (n = 150; metastasis-free survival: 68.1% at 10 years; overall survival: 79.9% at 10 years) (**Figures 1E and 1F**).

Because breast cancer is clinically and molecularly a heterogeneous disease, we selected a few representative breast cancer cell lines to sample this heterogeneity and explore the effects of a heterotypic culture by analyzing subtype-specific and shared response patterns. We focused on epithelial-osteoblast interactions, which were analyzed by co-cultivating NHOst cells with normal breast epithelial cells (HMECs) or 5 widely used breast cancer cell lines (MCF-7, T47D, SKBR-3, Hs578T and MDA-MB-231, each of which represents a different subtype of breast cancer).

The changes in gene expression that were due to heterotypic interactions were subtle when compared to the large intrinsic variation in expression patterns among the involved cell types, as illustrated in **Figure 1A** for the cell pairing of Hs578T and NHOst cells. To identify the gene expression changes that resulted from cell-cell interactions, it was necessary to control for the simple additive effects that reflect the proportional contribution of the two cell types to the total abundance of each gene's transcript in co-culture. Elimination of these proportionally weighted additive contributions allowed for the isolation of supra-additive interaction effects. The fact that the transcript levels of most genes did not change in response to co-culture allowed us to develop a linear regression model that was based on the transcriptional profiles of each monoculture and fitted to the co-culture data for normalization. For each gene, the ratio of the measured transcript level and the level that was estimated by the linear regression model provided a measure of the heterotypic interaction effect. Interaction effects, which are represented as gene-expression changes (induction or repression), were converted to quantitative values that can be analyzed for similarities and disparities over multiple different pair-wise interactions between two cell types with the same tools that are used to analyze conventional gene expression data, as described previously [18].

**Figure 2:**
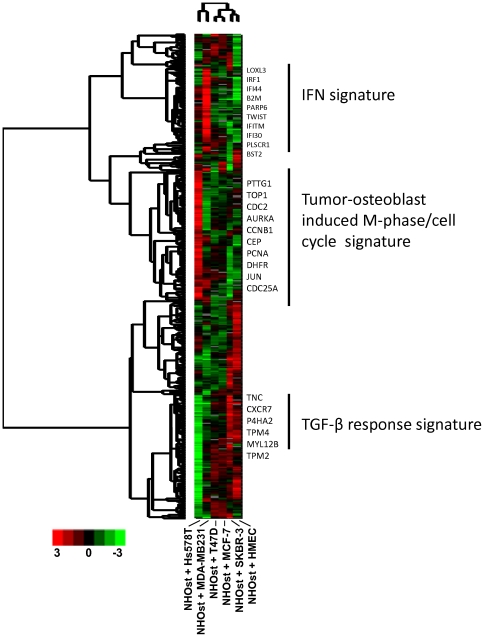
Gene expression changes in multiple co-cultures of breast cancer cell lines with osteoblasts. Overview of collapsed data from repeat co-culture experiments of six benign and malignant epithelial cell lines with NHOst cells. In a gene expression profiling experiment 7 monocultures and 6 co-cultures with NHOst were analyzed independently in duplicates. Genes with missing data in more than 20% of the arrays were removed, leaving 10774 gene IDs. Based on this dataset, the calculation of the interaction factors were performed separately for all co-cultures, as described in the Methods section. The interaction factors of the 6 co-cultures were further analyzed for their distribution, and factors with a standard deviation of <2 in at least two co-cultures were eliminated, leaving interaction factors for 635 genes, which are shown as a heat map (unsupervised hierarchical clustering). Red and green denote induction and repression due toheterotypic interaction. The magnitude of induction or repression is represented by color intensity. Specific clusters involved in IFN signaling, a “tumor-osteoblast cell-induced M phase/cell cycle” signature and response to TGF-β are marked.

There was obvious heterogeneity in the responses of the different pairs of cells to co-cultivation as shown by the heatmap of heterotypic interaction effects (**Figure 2**). Again, in this type of analysis which represented induction or repression of genes and not absolute expression values, a set of genes was shown to be induced in co-culture with Hs578T and NHOst that was not induced in co-culture with normal breast epithelial cells, HMECs, which confirmed the validity of this approach. To test for enrichment in a specific functional gene ontology, we applied the GO::TermFinder tool and found that a set of 37 genes which was highly enriched for genes associated with “cell division” (p = 0.00026) and the “M phase of the cell cycle” (p = 0.00016). This set of “tumor-osteoblast cell-induced M phase/cell cycle” genes included CCNB1, NCAPD2, CDC20, CCNA2, KIF20A, CDCA3, PTTG1, UBE2C, AURKA, DLGAP5, and KIF18A (**Figure S2**).

The HER2-positive breast cancer cell line, SKBR-3, in co-culture with NHOst cells induced the TGF-β gene and a set of TGF-β response genes (including up-regulation of the tropomyosins TPM1, TPM2, TPM4), supporting previous studies that have demonstrated the critical role of TGF-β signaling during formation of bone metastases [29]. Furthermore, an interesting interaction effect was found when we cultured MDA-MB-231 cells with NHOst cells. The MDA-MB-231 cells induced an interferon response with an up-regulation of characteristic interferon response genes, including 2′,5′-oligoadenylate synthetase 1 (OAS1), signal transducer and activator of transcription 1 (STAT1), IFIT, and interferon-induced transmembrane proteins (IFITMs). To analyze these effects in further detail, we focused on utilizing the co-culture of these two cell lines.

### Two specific gene sets are induced by the interaction between MDA-MB-231 cells with NHOst cells

**Figure 3:**
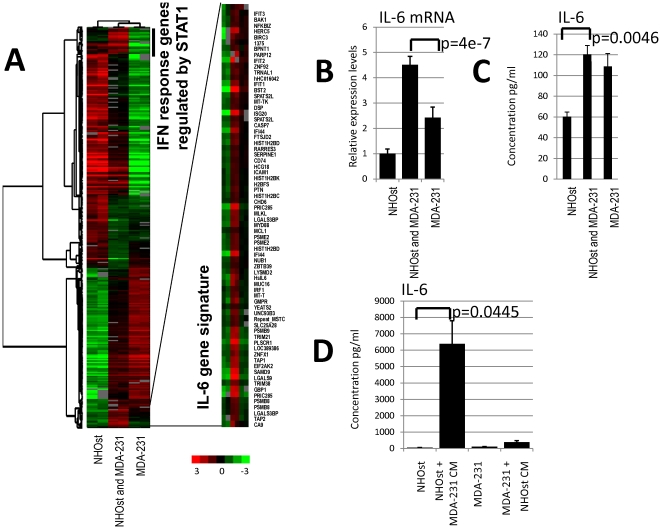
“Interferon response genes regulated by STAT1 signature” and “IL-6 gene signature” in co-culture. (**A**) Biologically independent replicates of the monocultured normal human osteoblasts (NHOst), the breast cancer cell line MDA-MB-231, and the mixed co-culture of NHOst and MDA-MB-231 cells were incubated for 48 hours under low serum conditions and characterized by DNA microarray hybridization. The figure shows the heat map of the hierarchical clustering of a total of 1461 elements that display a greater than 1.5-fold variance in expression of at least 2 different experimental samples. The co-culture of NHOst and MDA-MB231 induced two prominent sets of genes: An “interferon–response genes regulated by STAT1” signature and an “IL-6 gene signature” (zoomed image). (**B**) Real time PCR confirms a significantly higher expression of IL-6 mRNA in the co-culture than in either of the two monocultures (p = 4e-7; un-paired, two-tailed *t*-test). (**C**) As implied by the higher expression levels of IL-6 mRNA, the IL-6 concentration in the co-culture supernatants, as determined by ELISA, were significantly higher than the average concentration of the two monocultures. (p = 0.0046; un-paired, two-tailed *t*-test). (**D**) Also IL-6 was significantly more highly induced in NHOst cells stimulated with conditioned medium from MDA-MB-231 cells than in MDA-MB-231 cells stimulated with conditioned medium from NHOst (p = 0.045; un-paired, two-tailed *t*-test).

The interaction between MDA-MB-231 cells and NHOst cells in co-culture strongly induced two gene sets. In the first gene set, most of the induced genes have been identified to be regulated by interferon (**Figure 3A**), such as the genes OAS1, IFITM1, IFITM2, interferon response factor 9 (IRF9), β-2 microglobulin (B2M) and STAT1, which is the principal transcriptional regulator of the interferon response genes. In the monocultures, the expression of this gene set was higher in NHOst cells than in MDA-MB-231 cells. These genes were used to formulate a list of “interferon response genes regulated by STAT1” (**Table S1**). In the second gene set, which did not display much overlap with the first set, (2 genes only; MT-TK, SERPINE1, **Figure S3**), there was also up-regulation of interferon-regulated genes, such as IFI44 and IFIT1; genes related to apoptosis, such as caspase 7 (CASP7) and BCL2-antagonist/killer 1 (BAK); and proteasome genes, such as proteasome activator subunit 2 (PA28 beta), proteasome subunit beta type 8 (large multifunctional peptidase 7), and proteasome subunit beta type 9 (large multifunctional peptidase 2). A prominent gene of this set is interleukin-6 (IL-6), a cytokine with a wide variety of biological functions that is primarily involved in inflammation and the maturation of B cells [30]. A positive correlation between inflammation and cancer has been well established [31]. IL-6 has also been shown to be involved in the regulation of cell proliferation (in particular smooth muscle cell proliferation), the regulation of apoptosis and angiogenesis, and the positive regulation of osteoblast differentiation, all processes that might be involved in the development of bone metastases, as determined by SOURCE, a web-based database that brings together information from a broad range of resources, and provides it in manner particularly useful for genome-scale analyses [32]. Furthermore, there are genes that are known to be regulated by IL-6 through the STAT3 pathway, such as BST2 and pleiotrophin, which play a role in bone remodeling. Though both of the gene sets are up-regulated due to heterotypic interaction between MDA-MB-231 and NHOst cells, the genes of the first set were more highly expressed in NHOst cells (average expression: 0.036 above reference) than in MDA-MB-231 cells (average expression: −1.065 below reference); however, the genes of the second set were more highly expressed in the MDA-MB-231 cells (average expression: −0.104) than in the NHOst cells (−0.881). The set of genes that build the second cluster was used to formulate the “IL-6 gene signature” (**Table S2**).

### Increased IL-6 expression and cytokine levels in the co-culture of MDA-MB-231 and NHOst cells

To validate our gene expression data from the microarrays using an independent experimental approach, we examined the IL-6 mRNA expression levels with real-time quantitative PCR. For these experiments, we again co-cultured NHOst cells with MDA-MB-231 cells. In parallel, we cultured NHOst cells stimulated with the cell culture supernatant of MDA-MB-231 cells, and vice versa, for 24 hours in duplicate. Cell culture supernatants and RNA were isolated from each culture condition and IL-6 mRNA expression levels and protein concentration in the cell culture supernatant were analyzed. As shown in **Figure 3B**, the expression of IL-6 mRNA is significantly higher in the co-culture of MDA-MB-231 cells and NHOst cells than in either of the two monocultures (p = 4e-7; un-paired, two-tailed *t*-test). As implied by the higher expression levels of IL-6 mRNA, the IL-6 concentrations in the co-culture supernatant, as determined by ELISA, was significantly higher than the average concentration of the two monocultures. (p = 0.0046; un-paired, two-tailed *t*-test) (**Figure 3C**). Also IL-6 was significantly more induced in NHOst cells, which showed the lower IL-6 expression, when stimulated with conditioned medium from MDA-MB-231 cells than in MDA-MB-231 cells stimulated with conditioned medium from NHOst (p = 0.045; un-paired, two-tailed *t*-test) (**Figure 3D**).

### Effects of the “interferon response genes regulated by STAT1” and the “IL-6 gene signature” in primary breast cancer

**Figure 4:**
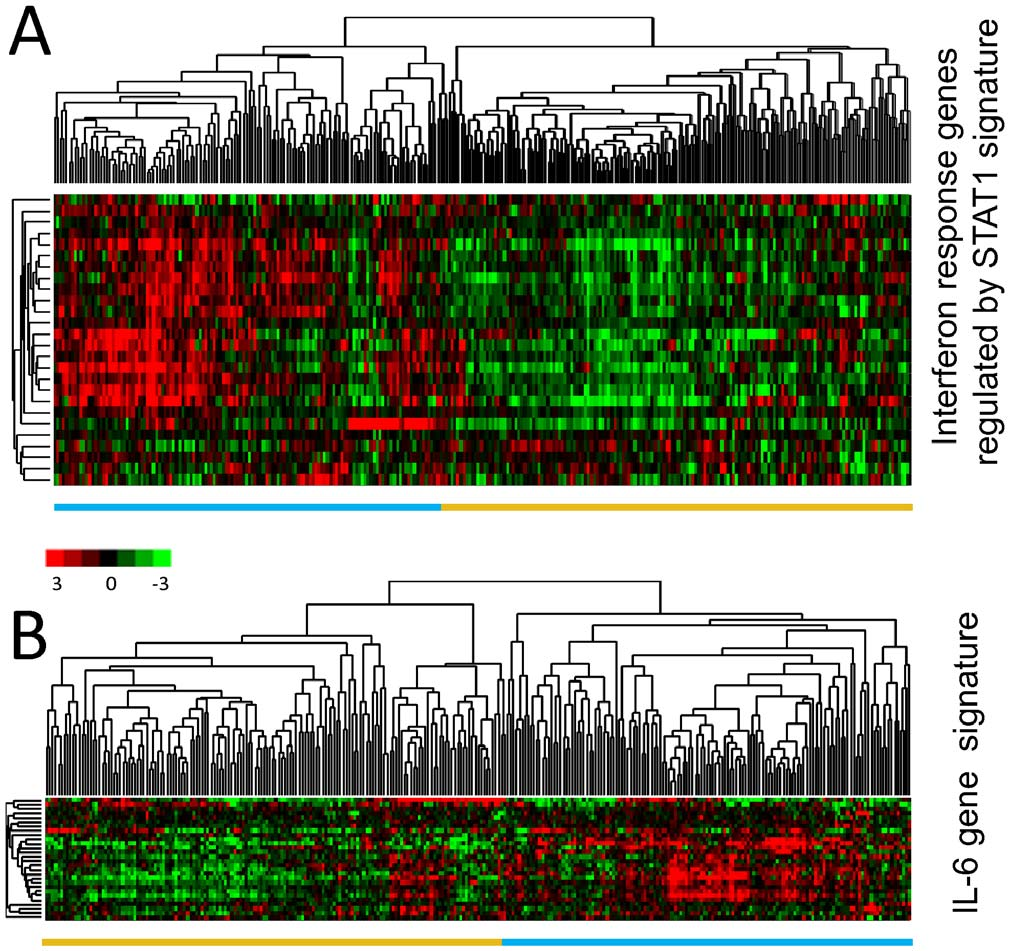
“Interferon response genes regulated by STAT signature” and “IL-6 gene signature” in early stage breast cancer. (**A**) The expression values of genes in the “interferon response genes regulated by STAT1 signature” were extracted from a published gene expression study of 295 early stage breast cancers from the Netherlands Cancer Institute [24]. Genes and samples are organized by unsupervised hierarchical clustering. The tumors segregated into two groups defined by high (blue bar below the heatmap) or low (golden bar below the heatmap) expression levels of 26 genes matching the “interferon response genes regulated by STAT1 signature”. (**B**) For the “IL-6 gene signature” an analogous analysis was performed, resulting in tumors segregated into two groups based on the expression levels of 28 genes. The two main clusters of tumors with high and low expression levels are marked with colored bars below the heatmap (blue and golden respectively).

**Figure 5:**
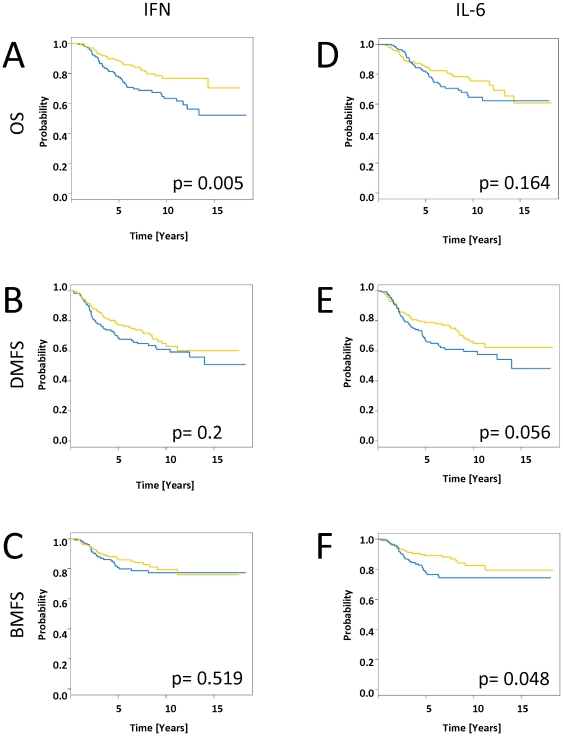
Effects of the “interferon response genes regulated by STAT1 signature” and “IL-6 gene signature” on bone metastasis formation. Kaplan-Meier plots for overall survival (OS) (A,D), distant metastasis-free survival (DMFS) (B,E) and bone metastasis-free survival (BMFS) (C, F) estimates are shown for the “interferon response genes regulated by STAT1 signature” and the “IL-6 gene signature”. P-values are given for the Cox regression analysis. The “IL-6 gene signature” segregated tumors with a significant difference in BMFS. The colors of the curves correspond to the colors of the bars below the heatmaps of figure 4 A and B.

To determine the effects of these two gene sets in primary tumors, we evaluated changes in their expression patterns in the published data from 295 early stage (stage I and II) breast cancer specimens from the Netherlands Cancer Institute (NKI) [24]. The “interferon response genes regulated by STAT1” showed a strikingly coherent variation in expression among these patients with cancer, which enabled them to be divided into two groups. We were able to cluster the breast carcinomas based only on the expression of the “interferon response genes regulated by STAT1” by separating them into two main clusters, with one cluster that displayed high-level expression levels of most of these genes and the other that displayed lower expression levels (**Figure 4A**). Distant metastasis-free survival and overall disease-specific survival were examined between the two groups. Early stage tumors with high expression levels (n = 136) of this particular gene set were characterized by lower distant metastasis-free survival (p = 0.2; 61% at 10 years) and significantly lower overall survival rates (p = 0.0046; 63% at 10 years) than tumors with low expression levels (n = 159; distant metastasis-free survival: 63% at 10 years; overall survival: 77% at 10 years) (**Figures 5A and B**).

The “IL-6 gene signature” also segregated the breast carcinomas of the NKI dataset in two groups, allowing the analysis of prognostic relevance (**Figure 4B**). This signature did not significantly correlate with overall survival (p = 0.164). However, interestingly, it displayed a trend toward formation of distant metastasis (p = 0.056) and was significantly associated with time to metastasis in the bone (as the primary site of metastasis; p = 0.048). Early stage tumors with high expression levels (n = 138) of this particular gene set had significantly reduced bone metastasis-free survival (74% at 10 years) than tumors with low expression levels (n = 157; metastasis-free survival: 83% at 10 years) (**Figures 5 D–F**). Interestingly, using the expression levels of the IL-6 gene alone we were unable to discriminate patients into two groups with a different amount of time to bone metastasis (p = 0.34, data not shown) as was the “interferon response genes regulated by STAT1” signature (**Figure 5C**).

### Correlations with other prognostic gene-expression signatures

**Figure 6:**
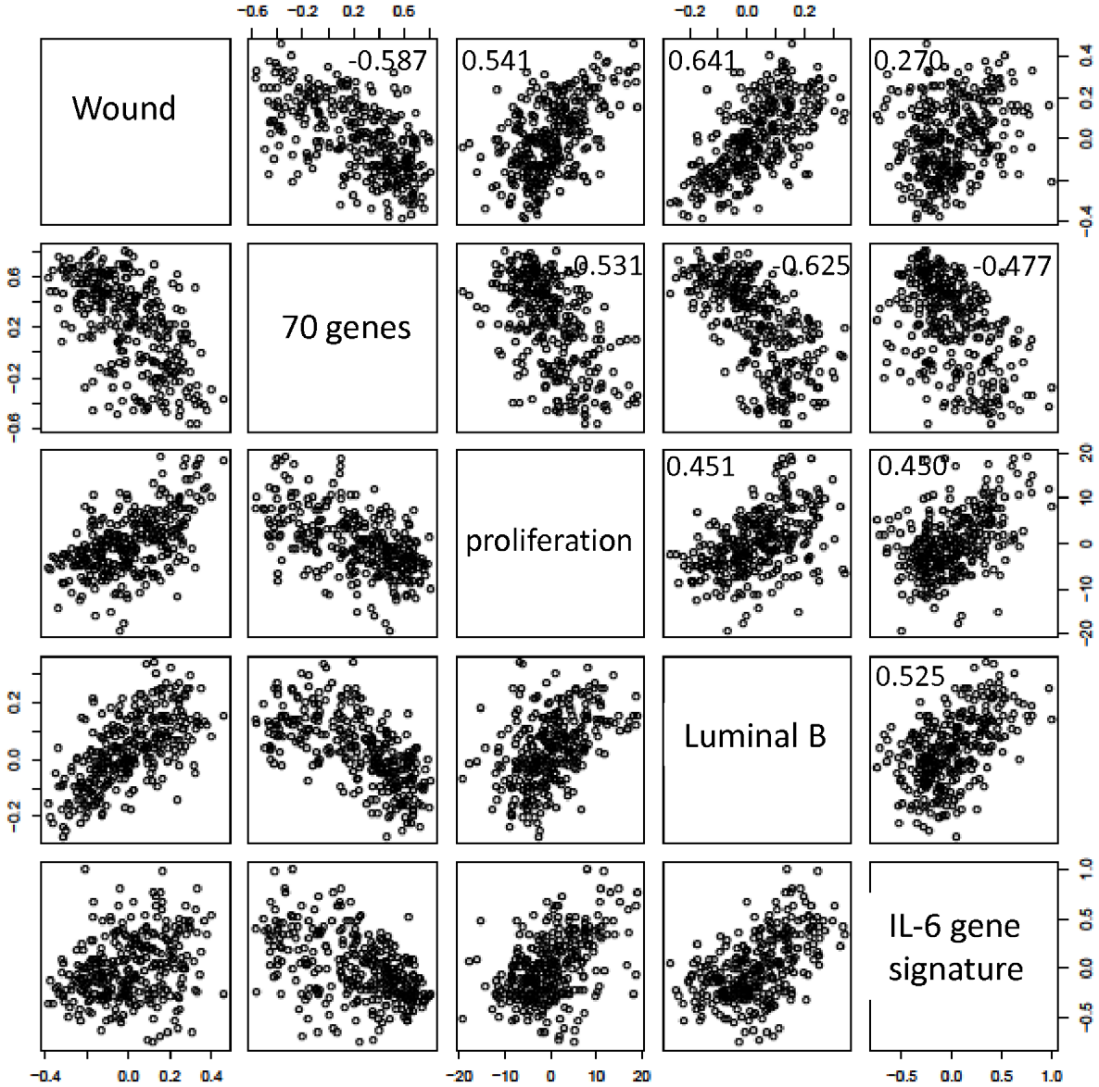
Correlations of the “IL-6 gene signature” with other published prognostic gene signatures in early stage breast cancer. Scatter plots showing the relationship between the value of the centroids of the “70-gene signature” [24], the “wound signature” [53], the “luminal type B signature” [26], the “proliferation” signature and the “IL-6 gene signature” in the NKI study. Each point in the scatter plots represents a single one of the 295 tumors analyzed in the NKI dataset. A 5×5 pair-wise scatter plot matrix of the five gene signatures is shown. Columns and rows are labeled in the diagonal panels, i.e. the first top right panel represents data for the centroids of the “Wound” signature plotted against the centroids of the “IL-6 gene signature”. The overall correlation between each pair of expression signatures across this set of 295 samples is indicated in each panel.

Because the signatures described above are prognostic markers in human breast cancer, we sought to determine whether they might be related to other previously published gene expression signatures that are useful prognosticators in the NKI dataset. Therefore, we correlated the signatures based on their centroids, which represent the average expression values of all genes building the signature in a single tumor specimen, using Pearson's correlation test. We investigated the relationship between the “IL-6 gene signature” or the “proliferation” signature and three previously identified gene expression signatures (**Figure 6**). The first signature is composed of 70 genes [33] and was identified by a supervised analysis of a subset of the NKI early stage breast cancer dataset [24]. This signature has been shown to predict freedom from metastasis at 5 years. The “IL-6 gene signature” and the “proliferation” signature did not correlate with the “70-gene” signature (r^2^ = −0.477 and −0.531). The second signature, known as the “wound signature,” was identified *in vitro* by exposing fibroblasts to serum to mimic a wound response, and it has been shown to predict the risk of breast cancer progression [34]. The “proliferation” signature correlated with the “wound signature” (0.541), whereas the “IL-6 gene signature” did not correlate well with the “wound signature” (0.269). The “proliferation” gene signature correlated with the luminal B profile, as defined by Soerlie et al. [26], with an r^2^ value equal to 0.451. The “IL-6 gene signature” also correlated with the luminal B profile, with an r^2^ value of 0.524. The detailed list of correlation values for all signatures that we analyzed may be found in **Table S3**. These results suggest that the “IL-6 gene signature” might add additional information to some of the formerly described signatures.

## Discussion

The main objective of this study was to examine and characterize the effects of heterotypic cellular interactions to gain insight into the diverse underlying biological mechanisms of skeletal metastasis of breast cancer, with a specific focus on the interaction between epithelial tumor cells and osteoblasts. Metastasis is a complex process that involves integrated activity of various genes that function at discrete steps, including angiogenesis, invasion, intravasation, survival in the circulation, extravasation, and homing and proliferation at the sites of metastasis [35]. To isolate specific, direct interactions from the more complex interactions that involve multiple cell types in a whole tissue or organism, we used a simple *ex vivo* co-culture model. Because certain important heterotypic interactions might require direct cell-cell contact, we focused on a co-culture model where the two cell types were mixed in equal proportion. In this report, we described the systematic genomic analysis of this simple *in vitro* system that simulates direct and indirect interactions between benign and malignant epithelial cells and osteoblast cells.

### Common gene sets induced by tumor-osteoblast co-cultures

As expected, based on our experience examining tumor-fibroblast [10] and tumor-endothelium interactions [18], the picture of heterotypic interaction effects from the survey of diverse tumor cells with osteoblasts is complex and reflects the different abilities of normal and malignant cells to send and respond to signaling molecules. Our data show that the effects of the tumor-osteoblast interactions differ among breast cancer cell lines that are representative of different breast cancer subtypes. This is not astonishing because it is well known that various breast cancer cell lines have different capacities to induce bone metastasis [36]. SKBR-3 cells, during their interaction with NHOst cells, induced a TGF-β response with up-regulation of the expression of genes such as TPM and others. This is in line with the previous results utilizing a transgenic model, in which a constitutively active TGF-β induced increased levels of metastasis in the bones of mice [29]. T47D cells induced TFF-1, which had previously been identified to be over-expressed in primary breast cancers that preferentially relapse to the bone [37]. TFF-1 also correlated with bone metastasis in an independent cohort *in vivo* [36].

A prominent theme in our co-culture experiments was the expression of a set of genes that was characteristic of the mitotic phase of the cell cycle, which we called the “tumor-osteoblast cell-induced M phase/cell cycle” gene expression signature. This observation of gene expression levels is consistent with the phenotypic features of the cells, which displayed a higher proliferation rate in their respective co-cultures. In our study, the mere co-existence of two cell types of different origin, such as the Hs578T breast cancer cells and NHOst osteoblast cells, appears to be sufficient to induce this response. Cooperate induction between cells of different lineages is well known from developmental biology studies, in which stem cells cooperate with their environmental cells to form the stem cell niche. This stem cell niche concept has also been introduced in cancer. Cells with stem cell characteristics have been prospectively isolated from breast cancer using CD44 and CD24 as markers, which are present on Hs578T and MDA-MB-231 cells. The stem cell-like cells with highly potent tumor initiating properties, as they were described by Al Hajj et al. [38], clearly exhibited the CD44+/CD24− signature but were also characterized by additional markers. Others have shown that CD44+/CD24− cell lines contain these tumor-initiating cells [39]–[41], but we did not specifically focus on these stem cell-like cells or explicitly select for them.

MDA-MB-231 cells, which have been shown to induce bone metastasis in a murine model system [42], displayed two prominent patterns of gene expression caused by the interaction with osteoblasts: a signature of “interferon response genes regulated by STAT1” (**Table S1**) and a signature associated with IL-6 expression, the “IL-6 gene signature” (**Table S2**). The microarray data of IL-6 could be confirmed by detecting an induction of IL-6 mRNA by real-time PCR and the IL-6 protein by ELISA in the co-culture. Furthermore, based on stimulation with conditioned media we have shown that IL-6 is mainly induced in the osteoblasts rather than in the MDA-MB-31 tumor cells. From this experiment we conclude that a secreted factor is sufficient to stimulate IL-6 induction, however we cannot exclude, that in addition direct cell-cell contact might influence expression of IL-6 in co-culture. IL-6 has been described to be involved in the stress response induced by the invasion of an osteoblastic matrix by breast cancer cells [43]. This cytokine is known to attract and activate osteoclasts [44] and likely contributes to the tumor-host microenvironment *in vivo*. In particular, IL-6 has been implicated in the pathogenesis of osteolysis associated with Paget's disease [45], Gorham-Stout syndrome [46], and multiple myeloma [47]. IL-6 levels in breast cancer patients have been found to correlate with the clinical stage of the disease as well as with the rate of recurrence [48]. High IL-6 serum levels in breast cancer patients have been identified as an unfavorable prognostic indicator. A similar observation has been made in colorectal cancer [49].

In breast cancer, IL-6 is known to activate STAT3, which has been shown to be an important signaling molecule that is associated with an unfavorable prognosis; in fact, the principal mechanism of STAT3 activation is via the IL-6/gp130/Jak pathway [50]. STAT3 also plays an essential role in stem cell biology. Mammospheres are useful and well-established models to study stem cell biology in breast cancer. Interestingly, mammospheres from lymph node invasive breast carcinoma tissues have been shown to express IL-6 mRNA at higher levels than mammospheres from matched non-neoplastic mammary glands [41]. In addition, IL-6 mRNA was detected only in basal-like breast carcinoma tissues, an aggressive breast carcinoma variant that displays stem cell features [51]. Confirming our results, the precursors of osteoblasts, mesenchymal stem cells, in co-culture with MDA-MB-231 cells also massively induced IL-6 levels [15]. This suggests a role of IL-6 in breast cancer stem cell biology. Considering the central role that bone plays in metastasis formation, eventually becoming a niche for breast cancer stem cells, one might imagine that intervention might cure more patients if these pathways would be specifically blocked. The bisphosphonate zolendronic acid, which significantly decreases bone morbidity, was tested in an adjuvant situation to prevent breast cancer relapse. The results were mixed, indicating that there might be a subgroup of patients that would benefit from this type of therapy. The effect of adjuvant zolendronic acid on overall survival appeared small at first, but we cannot exclude the possibility that certain subgroups might benefit. The “IL-6 gene signature,” which identified patients at the highest risk for bone metastasis, might be a method to more efficiently select patients for such an approach. Blocking IL-6 directly also has shown mixed results thus far. Genes of the “IL-6 gene signature” would be potential targets for such an approach. A gene signature, rather than a single molecule, might be a more powerful method for selecting the right patients to treat with a certain therapy. In our example, the “IL-6 gene signature,” in contrast to IL-6 alone, was of statistically significant prognostic value. This could eventually improve the cure rate of this specific breast cancer subgroup.

Prognostic and predictive factors have been well established in breast cancer, but less is known about which metastatic site will be affected. Kennecke et al. [52] linked breast cancer subtypes with the occurrence of metastasis at specific sites (brain, liver, lung, bone, distant nodal, and pleural/peritoneal). They identified luminal B type tumors as having the highest frequency of bone metastasis. This is interesting because the “IL-6 gene signature” mostly correlated with luminal B type tumors. A better prediction of the specific site of metastasis would improve surveillance and eventually prevent relapse. Relapse in the bone represents an incurable situation today, but timely detection could eventually prevent morbidity caused by bone metastasis.

Metastasis formation is a complex process involving multiple cell types. Thus far, we have modeled the interaction of tumor cells with osteoblasts. In our previous work, we studied the interaction of tumor cells with fibroblasts in the primary tumor microenvironment, which allowed us to define an interferon response gene set [10] and characterize tumor-endothelial cell interactions [18], thereby linking cancer cells that have stem cell characteristics (CD44+/CD24−) with the highly proliferative tumor phenotype. Therefore, we are now prepared to study the more complex interactions among more than two cell types in parallel, and our co-culture technique may allow us to further explore the more complex interactions among the multiple molecules that operate in these cells to orchestrate the process of cancer metastasis. Our studies suggest that *in vitro* modeling of specific processes and features of the tumor microenvironment can provide a valuable interpretive framework for the analysis of associated gene expression patterns in more heterogeneous *in vivo* samples and for the identification of the effects of heterotypic cellular interactions.

### Conclusion

In this study, we used breast cancer cell lines and osteoblasts in systematic co-culture experiments, which allowed us to characterize changes in gene expression. The gene signatures presented herein derived from the tumor-osteoblast co-culture might become promising predictors of the response to therapies for bone metastasis and could provide valuable hints about the role of IL-6 and its associated genes in bone metastasis formation.

## Supporting Information

Figure S1 **Graphical visualization of the output from GO::Termfinder for biological process ontology.** GOgraph layout that includes the significant GO nodes of the “interferon response genes regulated by STAT1 signature”, derived from 63 clones compared to a background of 922 clones. The colour of the nodes is an indication of their Bonferroni corrected P-value (orange < = 1e-10; yellow 1e-10 to 1e-8; green 1e-8 to 1e-6; cyan 1e-6 to 1e-4; blue 1e-4 to 1e-2; tan >0.01).
(TIF)

Figure S2 **Graphical visualization of the output from GO::Termfinder for biological process ontology.** GO-graph layout that includes the significant GO nodes of the “IL-6 gene signature”, derived from 62 clones compared to a background of 345 clones. The color of the nodes is an indication of their Bonferroni corrected P-value (orange< = 1e-10; yellow 1e-10 to 1e-8; green 1e-8 to 1e-6; cyan 1e-6 to 1e-4; blue 1e-4 to 1e-2; tan >0.01).
(TIF)

Figure S3 **Venn diagram depicting the overlap of the “interferon response genes regulated by STAT1 signature” (62 genes) and the “IL-6 gene signature” (63 genes).**
(TIF)

Table S1 **List of genes in the “interferon response genes regulated by STAT1 signature”.** The gene name, the gene symbol and the clone ID are listed.
(XLSX)

Table S2 **List of genes in the “IL-6 gene signature”.** The gene name, the gene symbol and the clone ID are listed.
(XLSX)

Table S3 **Correlations between the “70-gene signature”, the “wound signature”, the “luminal type B signature”, the “proliferation” signature and the “IL-6 gene signature” in the NKI dataset.** Summary of the r^2^ values describing the correlation between each pair of the 5 different gene expression signatures.
(TIF)
