# Supplementary figures and images for: Global Gene Expression Analysis of the Interaction between Cancer Cells and Osteoblasts to Predict Bone Metastasis in Breast Cancer
Source: PLoS One. 2012 Jan 3;7(1):e29743. doi: 10.1371/journal.pone.0029743 (PMC3250506; doi:10.1371/journal.pone.0029743)

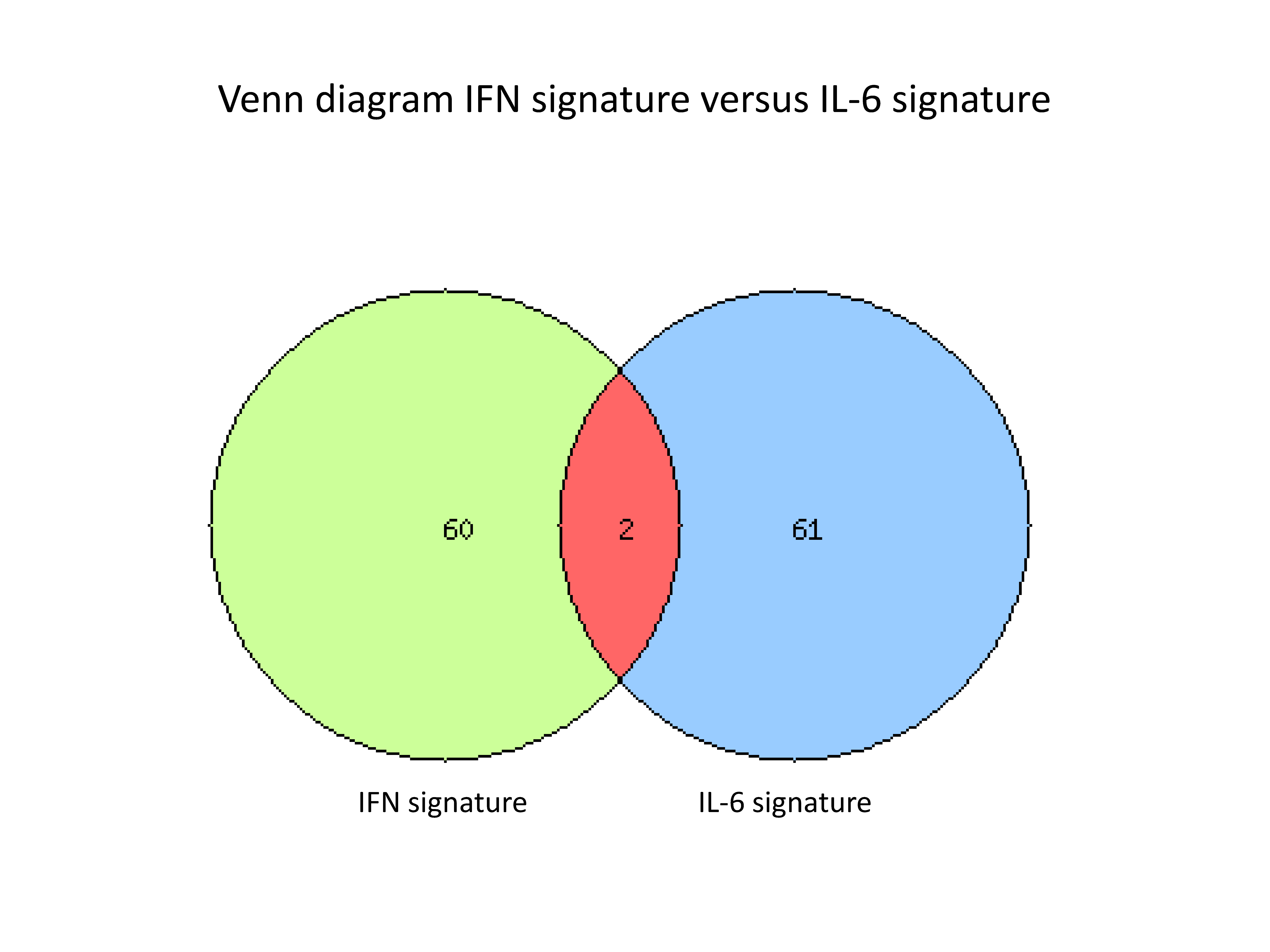

Supplement: Figure S3 — Venn diagram depicting the overlap of the “interferon response genes regulated by STAT1 signature” (62 genes) and the “IL-6 gene signature” (63 genes). (TIF) [file pone.0029743.s003.tif]
